# Effectiveness and Safety of Controlled Venous Pressure in Liver Surgery: A Systematic Review and Network Meta-Analysis

**DOI:** 10.1155/2015/290234

**Published:** 2015-05-13

**Authors:** Xue Liang Zhang, Wen Ji Wang, Wen Jin Wang, Nong Cao

**Affiliations:** ^1^Department of Orthopaedic, The First Hospital of Lanzhou University, Lanzhou 730000, China; ^2^Lanzhou University, Lanzhou 730000, China; ^3^Department of Emergency, The First Hospital of Lanzhou University, Lanzhou 730000, China; ^4^Department of General Surgery, The First Hospital of Lanzhou University, Lanzhou 730000, China

## Abstract

*Objective*. To investigate the effectiveness and safety of controlled venous pressure in liver surgery and further to compare the clinical outcomes of low central venous pressure by infrahepatic inferior vena cava clamping (IVCC) and intraoperative anesthetic control (IAC). *Methods*. Online databases including PubMed, Embase, Cochrane Library, Clinical trials.gov, and China biology medicine database were comprehensively searched. After identifying relevant studies out of the search results, quality assessment was performed according to the methods recommended by the Cochrane collaboration. And meta-analysis was performed by both direct comparison and indirect comparison. *Results*. Thirteen studies containing 1252 patients were included. Compared with control, controlled venous pressure significantly decreased central venous pressure, total blood loss, blood loss during transection, transfusion rate, and total incidence of complications. Further analysis of IVCC and IAC showed that there was no significant difference in aspects of main clinical outcomes. *Conclusions*. Controlled venous pressure significantly decreased central venous pressure and achieved improvement of bleeding control in liver surgery. It reduced total incidence of complications and chest infection, while it caused concerns about heart disorder. Although IVCC was not worse than IAC in therapeutic effect, a superiority between them still needs to be explored.

## 1. Introduction

As known, acute and long-term severe liver disease mainly including alcoholic and viral hepatitis can lead to an end-stage function failure. Besides, many uncontrolled chronic hepatitis patients will finally progress to be cirrhosis, which has a high risk to be malignant tumors [1]. For the treatment of function failure and tumors, liver transplantation and hepatectomy are regarded as the most curative ones [2]. It was reported that annually around 5700 liver transplantations were performed in Europe and 6000 cases in USA [3].

Nearly 14% of the whole blood is stored in liver, so liver surgery is always associated with high volumes of blood loss. In order to control bleeding from inflow system of liver blood supply, surgeons need to perform Pringle maneuver or selective vascular occlusion [4], and it is demonstrated to be effective. Meanwhile, some studies reported that a low central venous pressure (LCVP) also contributed an improvement in blood loss [5, 6], and this may control bleeding from outflow system of liver blood supply. Currently a LCVP was mostly achieved by intraoperative anesthetic control (IAC) and infrahepatic inferior vena cava clamping (IVCC). Compared with IAC, IVCC was considered to be easier to realize, and might to some extent ease concerns about abnormal homodynamic [7, 8].

Many clinical studies were designed to investigate the effectiveness and safety of controlled venous pressure in liver hepatectomy, liver resection, and transplantation [9]. However, the results were not completely consistent, and there were still no certain answers. Therefore, we performed a meta-analysis to define the therapeutic effect and safety of controlled venous pressure in liver surgery, and a network meta-analysis to further explore the difference between IAC and IVCC in clinical outcomes.

## 2. Materials and Methods

### 2.1. Search Strategy

Online databases including PubMed (1966.01–2014.12), Cochrane Library (2014 Issue 12), Embase (1974.01–2014.12), and China biology medicine database (up to 2014.12) were systematically searched. Search terms were (“infrahepatic inferior vena cava clamping” OR “IVC clamping” OR “IVCC” OR “low central vena cava pressure” OR “LCVP” OR “low central vein pressure”) AND (“liver surgery” OR “hepatic operation” OR “hepatectomy” OR “liver transplantation”). Medical subject headings, related articles function, and the references of reviews were also screened to widen the search results.

### 2.2. Literature Inclusion Criteria

The literature search results were firstly scanned by titles and abstracts, and eligibility for inclusion was further evaluated by reading full-texts by two reviewers independently (Wang Wen Ji, Zhang Xue Liang). Clinical studies investigating the method of controlled venous pressure in liver surgery were eligible. Patients with severe hepatic disease and who were willing to undergo a hepatectomy, liver resection, and transplantation were participants. All the characteristics of participants and treatment were comparable between treatment group and control group, except for the additional intervention of controlled venous pressure. The main outcome measures to evaluate therapeutic effect were total blood loss and blood loss during transection. Secondary outcome measures were transfusion rate, liver transection time, central venous pressure, and incidence of complications.

### 2.3. Data Extraction and Quality Assessment

Two reviewers (Zhang Xue Liang, Wang Wen Ji) extracted the information of the included studies, which mainly included author, publication year, group, cases, age, surgical procedures, and the data of outcome measures. Methodological quality of randomized controlled studies was assessed by using and modifying the methods recommended in the Cochrane handbook [10], which were based on six items: randomization, allocation concealment, blinding, comparative baseline, follow-up, and selective reporting. For high-quality comparative studies, Newcastle-Ottawa scale (NOS) was adopted to assess methodological quality, which was mainly based on patients selection, baseline comparability, and outcome measure [11]. Any disagreement about eligible and quality assessment were resolved through discussion or by a third reviewer (Cao Nong).

### 2.4. Statistical Analysis

Meta-analysis was conducted by using RevMan software (version 5.3, the Cochrane collaboration, Copenhagen, Denmark). The heterogeneity between studies was judged by Chi-square and *I* ^2^ statistical test. Random-effect model or fixed-effect model was chosen according to the heterogeneity test results. Pooled risk ratios (RR), mean difference (MD), and standard mean difference (SMD), with their 95% confidence intervals (95% CI), were presented for effect size. Network meta-analysis was conducted by using ITC soft (version 1.0, Canadian Agency for Drugs and Technologies in Health, Ottawa, Canada) [12]. The indirect comparison was handled and then a result in terms of statistical superiority/inferiority or no difference between the groups was assigned, and effect size with respective 95% CI was also presented.

This meta-analysis was conducted and reported mainly according to The Preferred Reporting Items for Systematic Reviews and Meta-Analyses: the PRISMA statement.

## 3. Results

### 3.1. Flow Diagram of Trial Selection

**Figure 1:**
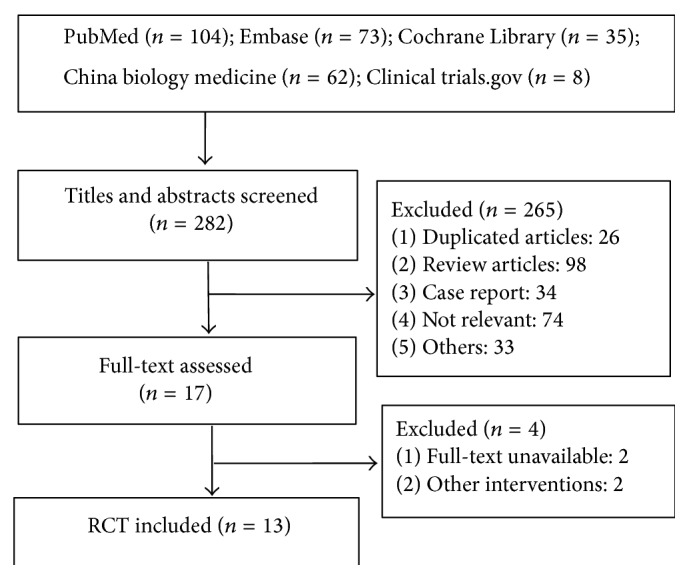
Flow chart of trial selection.

A total of thirteen trials [13–25] containing 1252 patients were included. There are 601 cases in the treatment group and 651 cases in the control group. Six of them [13, 14, 16, 18, 20, 23] compared IVCC with control, five of them [15, 17, 19, 24, 25] compared IAC with control, and two of them [21, 22] compared IVCC with IAC.  Figure 1 shows the flow chart from literature search result to final trial inclusion. The basic information of the included studies was extracted and described in Table 1. Nine of included studies were RCTs, and four of them were high-quality studies with more than 5 stars. The result of methodological quality assessment of included randomized controlled trials was shown in Table 2.

### 3.2. Meta-Analysis of Controlled Venous Pressure with Control

Compared with control, controlled venous pressure in liver surgery achieved a significant decrease in outcomes of central venous pressure [*I* ^2^ = 95%, MD = −2.67, 95% CI (−4.26, −1.09), *P* = 0.0009, Figure 3], total blood loss [*I* ^2^ = 95%, SMD = −0.81, 95% CI (−1.09, −0.54), *P* < 0.0001, Figure 4], blood loss during transection [*I* ^2^ = 84%, SMD = −0.99, 95% CI (−1.48, −0.50), *P* < 0.0001, Figure 5], and transfusion rate [*I* ^2^ = 2%, RR = 0.48, 95% CI (0.36, 0.64), *P* < 0.0001, Figure 6].

Further analysis of liver transection time indicated that there was no significant difference between the groups [*I* ^2^ = 9%, MD = −0.46, 95% CI (−1.68, 0.75), *P* = 0.45, Figure 7]. Controlled venous pressure also decreased total incidence of complications [*I* ^2^ = 0%, RR = 0.75, 95% CI (0.63, 0.91), *P* = 0.003, Table 3], and main diagnosis of complications was presented in Table 3, in which only chest infection finally reached a significant difference [*I* ^2^ = 0%, MD = 0.56, 95% CI (0.35, 0.90), *P* = 0.02].

### 3.3. Subgroup Analysis Comparing IVCC with Control

Compared with control, IVCC significantly reduced central venous pressure [*I* ^2^ = 94%, MD = −2.40, 95% CI (−4.13, −0.66), *P* = 0.007, Figure 3], total blood loss [*I* ^2^ = 68%, SMD = −0.64, 95% CI (−0.98, −0.31), *P* < 0.0001, Figure 4], blood loss during transection [*I* ^2^ = 91%, SMD = −1.01 95% CI (−1.80, −0.23), *P* = 0.01, Figure 5], transfusion rate [*I* ^2^ = 38%, RR = 0.46, 95% CI (0.32, 0.65), *P* < 0.0001, Figure 6], and total incidence of complications [*I* ^2^ = 0%, RR = 0.75, 95% CI (0.63, 0.91), *P* = 0.003, Table 3], whereas no significant difference was found in liver transection time [*I* ^2^ = 0%, MD = −0.24, 95% CI (−1.52, 1.04), *P* = 0.71, Figure 7].

### 3.4. Subgroup Analysis Comparing IAC with Control

Compared with control, IAC significantly decreased central venous pressure [MD = −4.00 95% CI (−4.60, −3.40), *P* < 0.0001, Figure 3], total blood loss [*I* ^2^ = 70%, SMD = −1.05, 95% CI (−1.52, −0.57), *P* < 0.0001, Figure 4], blood loss during transection [*I* ^2^ = 39%, SMD = −0.97, 95% CI (−1.44, −0.50), *P* < 0.0001, Figure 5], transfusion rate [*I* ^2^ = 0%, RR = 0.56, 95% CI (0.34, 0.91), *P* = 0.02, Figure 6], and total incidence of complications [*I* ^2^ = 0%, RR = 0.68, 95% CI (0.55, 0.86), *P* = 0.0009, Table 3]. There were also no significant differences in aspects of liver transection time [*I* ^2^ = 25%, MD = −2.36, 95% CI (−6.10, 1.37), *P* = 0.22, Figure 7].

### 3.5. Network Meta-Analysis of IVCC with IAC

**Figure 2:**
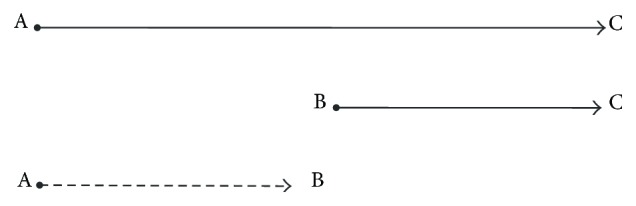
Network diagram of clinical trials according to the methods used to control venous pressure. A represented IVCC, B represented IAC, and C represented control.

**Figure 3:**
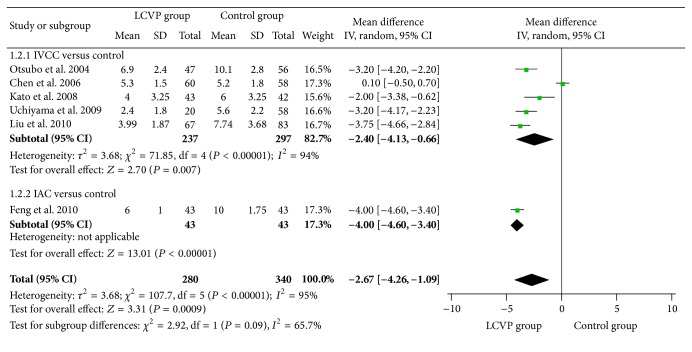
Meta-analysis of central venous pressure between LCVP and control.

**Figure 4:**
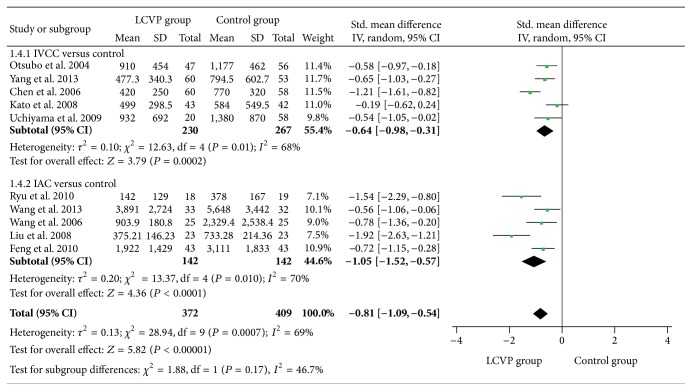
Meta-analysis of total blood loss between LCVP and control.

**Figure 5:**
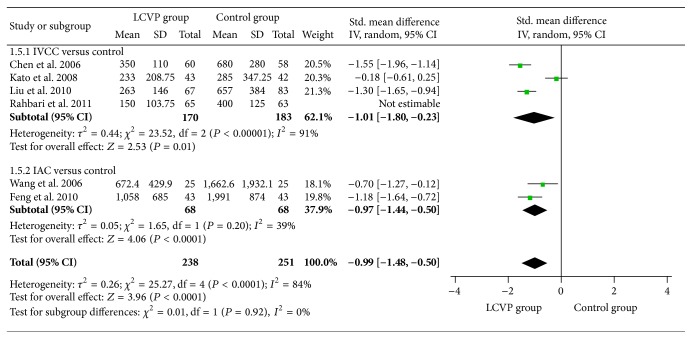
Meta-analysis of liver transected blood loss between LCVP and control.

**Figure 6:**
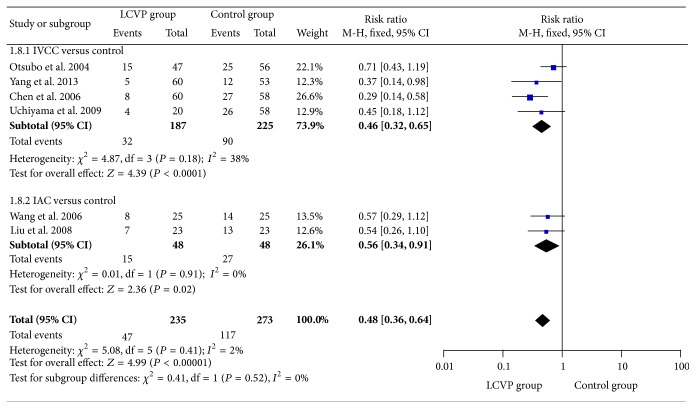
Meta-analysis of transfusion rate between LCVP and control.

**Figure 7:**
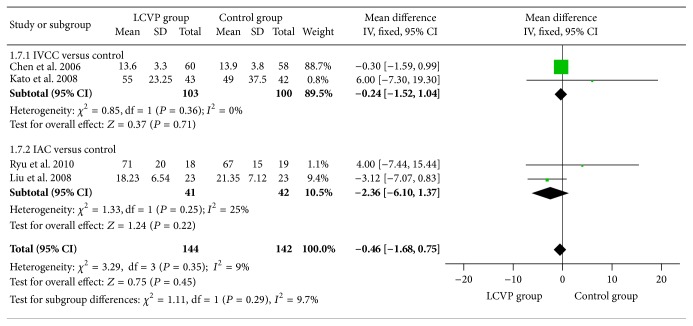
Meta-analysis of liver transected time between LCVP and control.

Due to insufficient numbers and cases of direct comparison, we also performed a network meta-analysis.  Figure 2 shows the network of clinical studies according to the methods used to control central venous pressure. Two studies compared IVCC with IAC; meta-analysis results indicated that IVCC significantly decreased total blood loss, blood loss during transection, total operation time, and liver transection time. However, the network analysis results showed that there were no significant difference in aspects of central venous pressure, total blood loss, total operation time, liver transection time, transfusion rate, and total complications, as shown in Table 4.

### 3.6. Publication Bias

**Figure 8:**
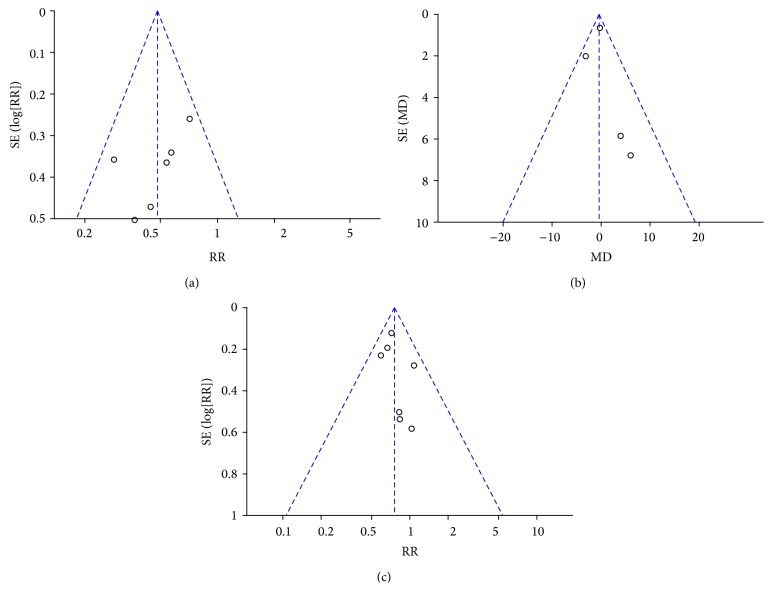
Funnel plot. (a) Transfusion rate; (b) liver transection time; (c) total complications.

Inverted funnel plots were adopted to evaluate publication bias. The results of transfusion rate, liver transection time, and total complications did not reveal asymmetry, indicating little possibility of publication bias (Figure 8).

## 4. Discussion

The meta-analysis included 13 high-quality studies comparing controlled central venous pressure with control in liver surgery, and it demonstrated a strong relationship between blood loss and outflow system of liver blood supply. Meanwhile, controlled venous pressure also had advantages in aspects of other clinical outcome measures.

There was a significant reduction of 2.67 cm H_2_O in central venous pressure, and meta-analysis of 10 trials in the random-effects model showed that the total blood loss was significantly reduced. To estimate the total blood loss, four of the analyzed studies collected blood in the container of the aspirator and weighed the soaked gauzes [14, 15, 17, 18], and additionally one study visually evaluated the surgical field [24], while the other studies did not report the methods, so this may be a source of heterogeneity across the studies. We further investigated the blood loss during liver transection in five studies, which was more accurate than total blood loss. Among the studies, four of them performed hepatectomy [14–16, 20] and one performed liver transplantation [19], and there was also a significant reduction in blood loss. In order to eliminate the influence of transection area in hepatectomy, one study [21] calculated the blood loss per transection area (mL/cm^2^), and it further demonstrated a significant difference between the groups (*P* = 0.01).

Further analysis revealed that the transfusion rate was also significantly decreased in IVCC and IAC group, while only one study presented a detailed protocol of transfusion. Although the transfusion protocols between the centers were different and it might cause a heterogeneity, it may not influence the pooled results as the protocols were comparable between the groups in each study. Therefore, combining all the results mentioned above, controlled central venous pressure though IVCC and IAC really significantly improved the intraoperative bleeding control and reduced the blood loss and thus induced a lower transfusion rate.

In aspects of other clinical outcomes, controlled venous pressure might also decrease operation time, while meta-analysis result of liver transection time revealed that there was no significant difference.

For safety, controlled venous pressure achieved a lower incidence of complications mainly in IAC subgroup, and further analysis of diagnosis only found a significant difference in chest infection. Many concerns about abnormal homodynamic did not have significant differences between the groups, perhaps due to the optimal intraoperative anesthetic control [22]. Liver function and renal function were monitored in each study, and relevant indexes including alanine transaminase (ALT), aspartate aminotransferase (AST), and blood urea nitrogen (BUN) were influenced, whereas they almost recovered to normal level in postoperative three days, while blocked venous blood from infrahepatic vena cava may induce some risks of incidence of heart disorder (RR = 1,36, *P* = 0.76), which might involve the opening of collateral circulation and increased blood and pressure in azygos vein from hemiazygos vein and accessory hemiazygos vein.

Further comparisons between IVCC and IAC were conducted using both traditional meta-analysis and network meta-analysis. There was no significant difference in aspects of center vein pressure reduction, transfusion rate, and total complications, while different results existed in aspects of blood loss and operation time. Meta-analysis based on two trials revealed that IVCC significantly decreased total blood, blood loss during transection, total operation time, and liver transection time. Network meta-analysis showed that there was no significant difference between the groups. Besides, compared with IAC, IVCC was really easy to realize as it only needs to clamp the IVC briefly with a vascular clamp before the actual transection [21, 22]. Although we cannot accurately define a superiority between them, current evidence strongly demonstrated that IVCC was not inferior to IAC in aspects of clinical outcome measures, and it still had distinct advantages in feasibility.

Limitations that existed in the meta-analysis were as follows. (1) For all the surgeries, surgeon's experience played important roles in clinical outcomes [26]. We could only ensure it was comparable in each study, and the difference between studies might be the most influencing factors and sources of heterogeneity, which could not be overcome. (2) Other factors such as surgical equipment, operation environment, and characteristics of participants were also different. Although comparable in each study, they also had some negative influence. (3) Although IVCC was not worse than IAC, the difference between them still needs to be explored and confirmed in future studies.

In conclusion, controlled venous pressure significantly decreased central venous pressure and achieved improvement of bleeding control in liver surgery. It reduced total incidence of complications, while it caused some concerns about heart disorder. Although IVCC was not worse than IAC in clinical treatment efficacy, a superiority between them still needs to be explored in the future.

## Figures and Tables

**Table 1:** Characteristics of included studies.

Study	Country	Case (T/C, *n*)	Age (T/C, y)	Sex (M/F)		Intervention	Baseline CVP (T/C)	Surgery	Diagnosis
				T	C				
Otsubo et al. 2004 [13]	Japan	47/56	59.3 ± 14.4/55.4 ± 16.1	30/17	41/15	IVCC/control	10.6 ± 2.6/10.1 ± 2.8	Hepatectomy	Hepatocarcinoma (28/31), metastatic tumor (10/14), cholangiocellular carcinoma (5/4), others (4/7)

Chen et al. 2006 [14]	China	60/58	39.7 ± 3.6/41.5 ± 4.1	53/7	51/7	IVCC/control	7.6 ± 3.0/7.7 ± 2.4	Hepatectomy	HBV related cirrhosis (51/54), HCV related cirrhosis (6/4), others (1/2)

Wang et al. 2006 [15]	China	25/25	45.3 ± 14.6/46.0 ± 12.1	19/6	21/4	IAC/control	—	Hepatectomy	Liver cirrhosis (14/15), others (11/10)

Kato et al. 2008 [16]	Japan	43/42	65 (28–82)/67 (38–79)^∗^	—	—	IVCC/control	7 (3–14)/7 (2–16)^∗^	Liver resection	Hepatocarcinoma (35/34), metastatic tumor (6/7), cholangiocellular (1/0)

Liu et al. 2008 [17]	China	23/23	45.4 ± 13.0/42.9 ± 12.6	14/9	16/7	IAC/control	2.7–5.4	Liver resection	Hepatocarcinoma (23/23)

Uchiyama et al. 2009 [18]	Japan	20/58	61.6 ± 12.9/64.8 ± 16.8	15/5	44/14	IVCC/control	—	Hepatectomy	Hepatocarcinoma (9/31), metastatic tumor (6/20), others (5/7)

Feng et al. 2010 [19]	China	43/43	48 ± 10/47 ± 12	6/37	8/35	IAC/control	10 (6–12)/10 (6–11)^∗^	Transplantation	Hepatocirrhosis (15/16), severe hepatitis (7/8), carcinoma (19/17), others (2/2)

Liu et al. 2010 [20]	China	67/83	51 (33–69)/55 (14–76)^∗^	48/35	55/12	IVCC/control	7.8 ± 3.5/8.3 ± 3.6	Liver lobectomy	Primary liver carcinoma (67/83)

Rahbari et al. 2011 [21]	Germany	65/63	57.2 ± 10.9/59.2 ± 12.1	37/28	42/21	IVCC/IAC	9.2 ± 3.6/7.6 ± 3.6	Liver resection	Hepatocarcinoma (19/20), metastatic tumor (35/37), others (11/6)

Zhu et al. 2012 [22]	China	96/96	—	—	—	IVCC/IAC	10.5 ± 1.3/10.8 ± 1.2	Liver resection	Liver tumors (96/96)

Yang et al. 2013 [23]	China	60/53	48.7 ± 10.8/49.5 ± 12.1	43/17	38/15	IVCC/control	7.6 ± 3.2/—	Hepatectomy	Hepatocarcinoma (31/26), metastatic tumor (7/7), others (22/20)

Ryu et al. 2010 [24]	South Korea	19/19	29 ± 11/26 ± 9	15/3	18/1	IAC/control	8.5/7.5	Liver lobectomy	Donors (19/19)

Wang et al. 2013 [25]	China	33/32	37.1 ± 10.1/35.9 ± 10.3	26/7	26/6	IAC/control	—	Liver lobectomy	Hepatocarcinoma (11/18), hepatocirrhosis (16/12), hepatitis B (4/1), others (2/1)

IVCC: infrahepatic inferior vena cava clamping; IAC: low central vein pressure by intraoperative anesthetic control; T: treatment group; C: control group.

^∗^Data were presented as median (range).

**Table 2:** Quality assessment of included RCTs.

Study	Randomization	Blinding	Allocation concealment	Comparative baseline	>80% follow-up	Free of selective reporting
Chen et al. 2006 [14]	Mentioned	Unclear	Unclear	Yes	Yes	Yes
Wang et al. 2006 [15]	Yes	Yes	Yes	Yes	Yes	Yes
Kato et al. 2008 [16]	Yes	Unclear	Unclear	Yes	Yes	Yes
Liu et al. 2008 [17]	Mentioned	Yes	Unclear	Yes	Yes	Yes
Feng et al. 2010 [19]	Yes	Yes	Yes	Yes	Yes	Yes
Rahbari et al. 2011 [21]	Yes	Yes	Yes	Yes	Yes	Yes
Zhu et al. 2012 [22]	Yes	Yes	Yes	Yes	Yes	Yes
Ryu et al. 2010 [24]	Yes	Yes	Yes	Yes	Yes	Yes
Wang et al. 2013 [25]	Yes	Yes	Yes	Yes	Yes	Yes

Yes: the method was properly adopted and used; mentioned: the method was reported without detailed description; unclear: no relevant information was found.

**Table 3:** Subgroup analysis of complications between LCVP and control group.

Subgroup	Study	Case (*n*/*N*)		Heterogeneity		Effect size	
		LCVP	Control	*I* ^2^	P	RR(95% CI)	P
Diagnosis							
Chest infection	[14, 18–20, 23, 25]	20/283	37/327	0%	0.96	0.56 (0.35, 0.90)	0.02
Pleural effusion	[14, 18–20, 23, 25]	41/283	57/327	43%	0.12	0.72 (0.51, 1.02)	0.06
Wound infection	[14, 23]	5/120	7/111	0%	0.75	0.64 (0.21, 1.93)	0.43
Ascites	[14, 18, 23]	12/140	22/169	0%	0.81	0.56 (0.29, 1.08)	0.08
Heart disorder	[18, 20]	1/87	2/141	0%	0.40	1.36 (0.19, 9.67)	0.76
Bile leakage	[18, 20]	2/80	2/111	0%	0.75	1.41 (0.22, 9.23)	0.72
Bleeding	[18, 20]	0/80	3/111	0%	0.45	0.35 (0.05, 2.67)	0.31
Hepatic insufficiency	[18, 20]	3/80	5/111	0%	0.49	0.90 (0.21, 3.85)	0.88
Sepsis	[18, 20]	3/103	4/101	0%	0.33	0.77 (0.20, 3.02)	0.71
Methods							
IVCC versus control	[14, 18, 20, 23]	52/207	69/252	0%	0.50	0.83 (0.62, 1.11)	0.2
IAC versus control	[15, 19, 25]	47/101	68/100	0%	0.70	0.68 (0.55, 0.86)	0.009
Total							
LCVP versus control	**[14, 15, 18–20, 23, 25]**	**99/308**	**137/352**	**0%**	**0.71**	**0.75 (0.63, 0.91)**	**0.003**

**Table 4:** Network analysis results between IVCC and IAC.

Outcomes	Direct comparison		Indirect comparison	
	MD (95% CI)	*P*	MD (95% CI)	GRADE
Central venous pressure	0.76 (−1.17, 2.70)	0.05	1.6 (−0.24, 3.44)	High
Total blood loss	−346.0 (−423.89, −268.12)	<0.0001	180.81 (−72.4, 433.66)	Low
Blood loss during transection	−249.22 (−288.84, −209.61)	<0.0001	677.69 (325.73, 1029.65)	Low
Operation time	−10.0 (−18.73, −1.28)	0.02	5.02 (−11.26, 21.3)	Moderate
Liver transected time	−2.17 (−3.23, −1.11)	<0.0001	2.12 (−1.62, 5.86)	Low

	RR (95% CI)	*P*	RR (95% CI)	Grade

Transfusion rate	0.83 (0.52, 1.32)	0.44	0.82 (0.45, 1.51)	High
Total complications	0.97 (0.77, 1.23)	0.80	1.22 (0.85, 1.76)	High

GRADE: Grading of Recommendations, Assessment, Development, and Evaluation.
